# Reviewer acknowledgement 2012

**DOI:** 10.1186/1710-1492-9-3

**Published:** 2013-03-26

**Authors:** Richard Warrington

**Affiliations:** 1 University of Manitoba, Manitoba, Canada

## Abstract

**Contributing reviewers:**

The editors of *Allergy, Asthma & Clinical Immunology* would like to thank all of our reviewers who have contributed to the journal in Volume 8 (2012).

## 

Catarina Almqvist

Sweden

Sami Bahna

United States of America

Allan Becker

Canada

Sowmya Bharani

United Kingdom

Miguel Blanca Gómez

Spain

Abraham Bohadana

Canada

Knut Brockow

Germany

Dominique M Bullens

Belgium

Scott Cameron

Canada

Stuart Carr

Canada

Timothy Caulfield

Canada

Edmond Chan

Canada

Don Cockcroft

Canada

Philip Cooper

United Kingdom

Pim de Boer

Netherlands

Pascal Demoly

France

Tony Dubois

Netherlands

Anne Ellis

Canada

Chandra Ghosh

United States of America

Paul Greenberger

United States of America

Eyal Grunebaum

Canada

Elizabeth Hazel

Canada

Ingmar Heijnen

Switzerland

Bo Huang

China

Timo Hugg

Finland

Rhoda Kagan

Canada

Alan Kaplan

Canada

Nader Khalidi

Canada

Khashayarsha Khazaie

United States of America

Harold Kim

Canada

Alex KleinJan

Netherlands

Sharon Kling

South Africa

Rajesh Kumar

United States of America

Christine Lejtenyi

Canada

Chantal Lemire

Canada

Sean Mace

Canada

Zubin Master

United States of America

Bruce Mazer

Canada

Christine McCusker

Canada

Rachel McLoughlin

Ireland

Michel Moutschen

Belgium

Thomas Nutman

United States of America

Cevdet Özdemir

Turkey

Jeremy Parker

United Kingdom

Linda Saif

United States of America

Carrock Sewell

United Kingdom

Fanny Silviu-Dan

Canada

John Simpson

United Kingdom

Agnes Sonnenschein-van der Voort

Netherlands

Gordon Sussman

Canada

Francesco Tovoli

Italy

Antonio Tristano

Spain

Stuart Turvey

Canada

Rubén Vicente García

Spain

Peter Villiger

Switzerland

Cynthia Visness

United States of America

Susan Waserman

Canada

